# Prevalence of *Salmonella* in broiler chickens in Kagoshima, Japan in 2009 to 2012 and the relationship between serovars changing and antimicrobial resistance

**DOI:** 10.1186/s12917-019-1836-6

**Published:** 2019-04-08

**Authors:** Vu Minh Duc, Yuko Nakamoto, Ayaka Fujiwara, Hajime Toyofuku, Takeshi Obi, Takehisa Chuma

**Affiliations:** 1 Laboratory of Veterinary Public Health, Joint Faculty of Veterinary Medicine, Kagoshima University, 1-21-24 Korimoto, Kagoshima, 890-0065 Japan; 2 The United Graduate School of Veterinary Science, Yamaguchi University, 1677-1 Yoshida, Yamaguchi, 753-8515 Japan; 3 Laboratory of Veterinary Microbiology, Joint Faculty of Veterinary Medicine, Kagoshima University, 1-21-24 Korimoto, Kagoshima, 890-0065 Japan

**Keywords:** *Salmonella*, Antimicrobial resistance, Serovar, Prevalence, Broiler, Beta-lactam, Cephalosporin, Fluoroquinolone

## Abstract

**Background:**

This study aimed to examine the prevalence, serovars, and antimicrobial resistance of *Salmonella* isolates from broiler chickens in Kagoshima, Japan. A total of 192 flocks and 3071 samples were collected from broiler chickens at local farms in Kagoshima, Japan from 2009 to 2012.

**Result:**

Among the tested farms, 49.0% of flocks were positive for *Salmonella*, and 243 isolates were obtained from 3071 cecal samples (7.9%). All the *Salmonella* isolates were one of three serovars: *S.* Infantis (57.6%); (140/243), *S.* Manhattan (40.3%; 98/243 and *S.* Schwarzengrund (2.1%; 5/243). The proportion of *S.* Infantis isolates decreased from 66.0% in 2009 to 50.0% in 2011 but increased to 57.6% in 2012, while the proportion of *S.* Manhattan isolates significantly increased from 26.4 to 50% from 2009 to 2011, and decreased moderately to 40.9% in 2012. Most of the recovered *Salmonella* isolates were resistant to three antimicrobials, i.e., streptomycin (95.1%), sulfamethoxazole (91.0%) and oxytetracycline (91.4%). In contrast, all *Salmonella* strains were susceptible to chloramphenicol. Comparison of this study to previous studies of the antimicrobial susceptibility of *Salmonella* isolates showed that: the percentage of antibiotic-resistance isolates increased dramatically for two antibiotics, ampicillin (from 22.4 to 55.1%) and cefotaxime (from 9.1 to 52.7%). In contrast, the percentage of ofloxacin-resistant isolates decreased across the three survey periods, from 20.8% in 2004–2006 to 1.6% in the present study period (2009–2012). In addition, *S.* Infantis exhibited a variety of resistance to antimicrobials examined from sensitive to resistance to eight antimicrobials. Multidrug resistance to more than 6 six antimicrobials was detected in 113 (46.5%) of the isolates, and most of them were *S.* Manhattan.

**Conclusions:**

There was a marked change in the serovars and antimicrobial resistance profiles of the *Salmonella* isolates in this study compared to those in previous studies. The percentage of *S.* Manhattan isolates increased as did the percentages of ampicillin- and cefotaxime-resistant isolates.

## Background

*Salmonella* is a major foodborne pathogen that causes an estimated 153 million enteric infections and 56,969 diarrheal deaths each year worldwide [1]. Chicken meat and eggs have been reported as a major source of *Salmonella* contamination. Therefore, it is important to control *Salmonella* in chicken- and egg-containing food products [2, 3].

Despite significant improvements in technology and hygienic practices at all stages of chicken production, salmonellosis and *Salmonella* infections remain an intransigent threat to human and animal health. In many countries the high incidence of salmonellosis in humans appears to be caused by infection derived from contaminated eggs, poultry meat and meat-containing products. The contaminated products cause disease as a result of inadequate cooking or cross contamination of working surfaces in the kitchen environment ([4, 5]. According to food poisoning statistics from the Infectious Disease Surveillance Center in Japan, there were 93,444 bacterial foodborne illnesses between 1999 and 2002, and 32% of these cases were salmonellosis (http://idsc.nih.go.jp/iasr/index.html). According to another survey in Japan, *Salmonella* is the second most common (after *Campylobacter* infection) cause of bacterial foodborne outbreaks [6].

Poultry, especially broiler chickens, are well known reservoirs of various *Salmonella* serovars, many of which are able to infect humans and *Salmonella* Infantis has been the most prevalent serovar isolated from fresh poultry meat and broiler flocks all over in Europe [7]. Nine serovars of *Salmonella* were detected in Japan from retail chicken meat in 2012, *S.* Infantis (33%), *S.* Schwarzengrund (12%), and *S.* Manhattan (9%) were the most frequent [8]. Beside, *Salmonella* Schwarengrund is one of the *Salmonella* serovars responsible for human and poultry infections in some countries, for sample, the United States, Denmark and Thailand [9, 10].

Antimicrobial resistance is becoming an increasingly important issue in salmonellosis in both animals and human [11]. In poultry production, antimicrobial agents are widely used for growth promotion, or treatment purposes [12]. As a consequence, chicken and chicken meat can harbor antimicrobial resistant strains and function as a vehicle for dissemination of these to human. Today, antimicrobial resistant of *Salmonella* strains are frequently encountered in most of the world and the proportion of antimicrobial resistant dramatically increased over the past decade [13].

Research on the epidemiology of *Salmonella* throughout the food chain is important for determining the specific distribution patterns of antimicrobial resistance for this pathogen. In this study, we analyzed the prevalence, serovars, and antimicrobial resistance profiles of *Salmonella* isolates from broiler chickens in Kagoshima, Japan. This knowledge will help to understand the relationship between changes in the serovar and antimicrobial resistance patterns of *Salmonella*, and define guidelines for improved salmonellosis control which in turn might lead to fever human foodborne salmonellosis cases.

## Results

### Prevalence and serovars of Salmonella isolated from broiler chickens in Kagoshima, Japan, in 2009–2012

The prevalence of *Salmonella* in broiler chickens in 2009–2012 in Kagoshima, Japan is presented in Table 1. The prevalence of *Salmonella-*positive flocks varied slightly from year to year during the study period, and the overall percentage of positive flocks was 49.0% (94/192). The same number of flocks (48 flocks) was collected each year. The percentage of positive flocks was 50.0% in 2009, which decreased dramatically to 39.6% in 2010. However, the trend then changed, and the percentage increased in the next two years to 45.8% in 2011 and 60.4% in 2012. However, there was no significant difference year by year from 2010 to 2012.

**Table 1:** Prevalence of *Salmonella* in broilers in Kagoshima, Japan in 2009–2012

Year	No. of flocks	No. of positive flocks (%)	No. of samples	No. of positive samples (%)
2009	48	24 (50.0)	768	53 (6.9)
2010	48	19 (39.6)	768	60 (7.8)
2011	48	22 (45.8)	767	64 (8.3)
2012	48	29 (60.4)	768	66 (8.6)
Total	192	94 (49.0)	3071	243 (7.9)

No.: Number

The prevalence of *Salmonella* among all tested samples was 7.9% (243/3071), and is was highest in 2012, at 8.6%, followed by 8.3% in 2011, 7.8% in 2010, and 6.9% in 2009. These differences were not significant.

**Fig. 1:**
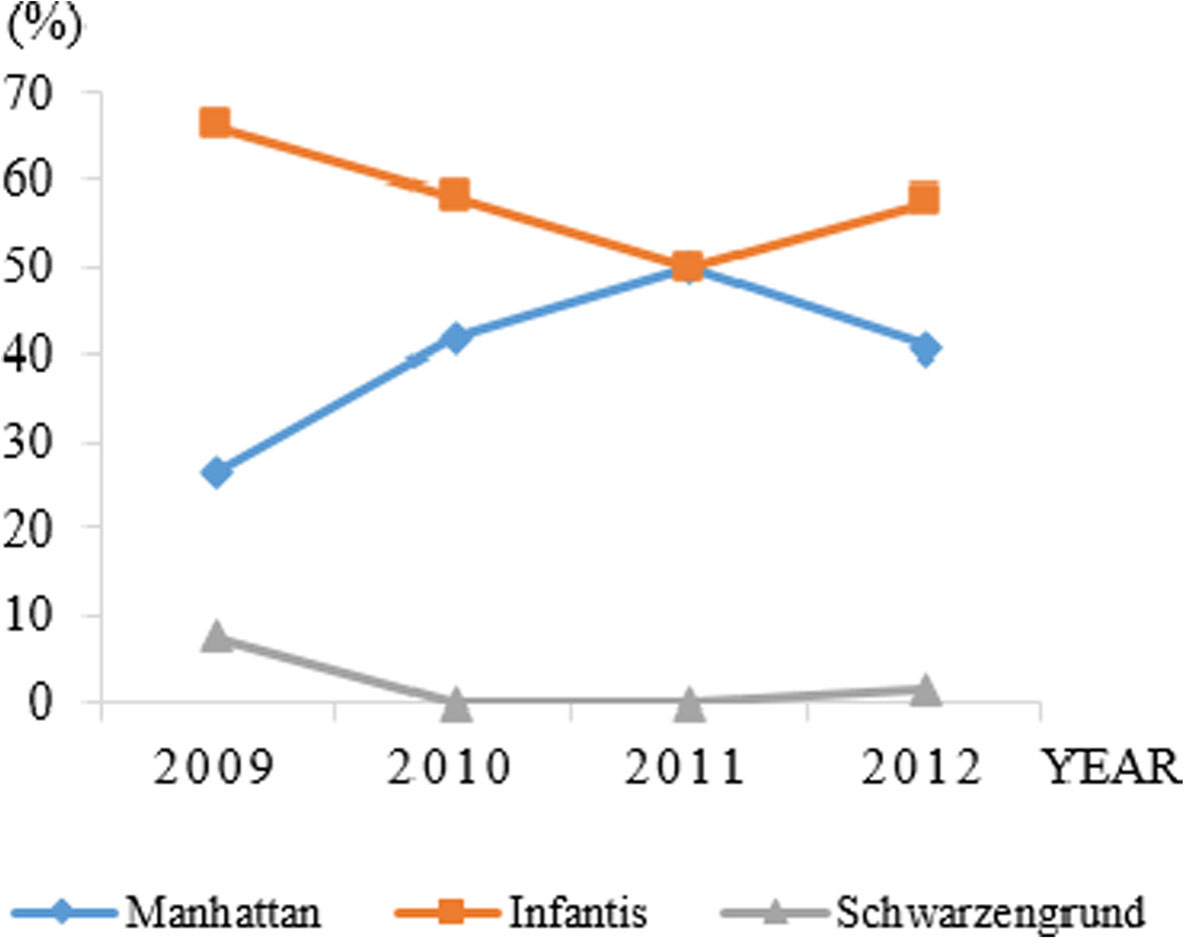
Change of distribution of *Salmonella* serovars isolates from broiler in Kagoshima, Japan in the period from 2009 to 2012

The year-to-year changes in the serovars of the *Salmonella* isolates were investigated, and the results are presented in Fig. 1. The strains of *Salmonella* isolated from broiler chickens in Kagoshima, Japan (*n* = 243) in the four year period in 2009–2012 belonged to three serovars, *S.* Infantis 57.6% (140/243), *S.* Manhattan 40.3% (98/243), and *S.* Schwarzengrund 2.1% (5/243).

Figure 1 shows a contrasting trend in the number of *S.* Manhattan and *S.* Infantis isolates detected from 2009 to 2012. The percentage of *S.* Infantis isolates decreased gradually, from 66.0% in 2009 to 50.0% in 2011, but increased to 57.6% in 2012, whereas the percentage of *S.* Manhattan isolates significantly increased from 26.4% in 2009 to 50.0% in 2011 after decreased moderately to 40.9% in 2012.

The highest percentage of *S.* Schwarzengrund isolates was observed in 2009 (7.6%; 4/53). This serovar was not detected in 2010 or 2011; however 1 isolate (1/66; 1.5%) was detected in 2012.

### Antimicrobial resistance phenotypes

The results of the MIC analysis of 243 *Salmonella* isolates are summarized in Table 2. All 243 strains were susceptible to chloramphenicol, with a MIC ≥32 μg/mL. The rates of resistance were the highest for streptomycin, sulfamethoxazole, and oxytetracycline, and > 90% of strains were resistant to these antimicrobials; 231 (95.1%) were resistant to streptomycin (MIC ≥ 16 μg/mL), 221 (91.0%) were resistant to sulfamethoxazole (MIC ≥ 512 μg/mL), and 222 (91.4%) were resistant to oxytetracycline (MIC ≥ 16 μg/mL). Many isolates were also resistant to ampicillin (55.1%) and cefotaxime (52.7%). The three antimicrobials with the lowest resistance rates were kanamycin (6.6%), cefoxitin (6.2%), and ofloxacin (1.6%).

**Table 2:** Antimicrobial susceptibility profiles of 243 *Salmonella* isolates from 2009 to 2012

Antimicro-bial agent	No. of isolates at the MIC (μg/mL)												MIC break-point (μg/ml)	Resistance no. (%)
	0.25	0.5	1	2	4	8	16	32	64	128	256	512		
SM	0	0	0	0	3	9	7	124	100	0	0	0	≥16	231 (95.1)
OTC	0	2	12	7	0	0	0	0	2	114	106	0	≥16	222 (91.4)
SUL	0	0	2	1	3	9	6	1	0	0	0	221	≥512	221 (91.0)
AMP	0	33	70	6	1	0	0	0	5	8	1	120	≥32	134 (55.1)
CTX	112	0	1	2	9	13	87	17	1	1	0	0	≥4	128 (52.7)
CTF	2	50	62	1	3	11	25	8	64	10	7	0	≥8	125 (51.4)
KM	15	0	17	144	53	0	0	0	0	0	0	16	≥64	16 (6.6)
CFX	0	2	45	114	60	2	5	10	5	0	0	0	≥32	15 (6.2)
OFLX	228	2	7	4	0	0	0	0	0	0	0	0	≥2	4 (1.6)
CP	0	32	95	110	4	0	0	0	0	0	0	0	≥32	0 (0.0)

*AMP* ampicillin, *CTX* cefotaxime, *CFX* cefoxitin, *CP* chloramphenicol, *SM*,streptomycin, *SUL* sulfamethoxazole, *OTC* oxytetracycline, *KM* kanamycin, *OFLX*,ofloxacin, *CTF* ceftiofur

Each serovar of *Salmonella* showed a different resistance prevalence to antibiotics used in the study (Table 3). Serovar *S.* Infantis and *S.* Manhattan exhibited resistance to streptomycin, sulfamethoxazole and oxytetracycline, ranging from 84.6% (resistance of *S.* Infantis to sulfamethoxazole) to 96.9% (resistance of *S.* Manhattan to streptomycin and oxytetracycline), while they were sensitive ofloxacin with a resistance rate of 1.0 and 2.1% in *S.* Manhattan and *S.* Infantis, respectively. We also found a significant difference in the antibiotic resistance rates of the two serovars to four other antibiotics. Resistance to cefoxitin, ceftiofur, cefotaxime and ampicillin was 0.0, 92.9, 93.9 and 94.9%, respectively, for *S.* Manhattan, and 10.7, 25.0, 25.7 and 29.3%, respectively, for *S*. Infantis. *S.* Schwarzengrund showed high sensitivity to seven antibiotics with 0% resistance, but exhibited a high resistance (100%) to streptomycin, sulfamethoxazole and oxytetracycline (Table 3).

**Table 3:** Distribution of antimicrobial resistance of Salmonella isolated from 2009 to 2012 according to the serovar

2009–2012 (No. Isolates)	SM (%)	OTC (%)	SUL (%)	AMP (%)	CTX (%)	CTF (%)	KM (%)	CFX (%)	OFLX (%)	CP (%)
Infantis (*n* = 140)	132 (94.3)	122 (87.9)	121 (86.4)	41 (29.3)	36 (25.7)	34 (25.0)	13 (8.6)	15 (10.7)	3 (2.1)	0(0.0)
Manhattan (*n* = 98)	94 (95.9)	95 (96.9)	95 (96.9)	93 (94.9)	92 (93.9)	91 (92.9)	3 (2.0)	0 (0.0)	1 (1.0)	0 (0.0)
Schwarzengru-nd (*n* = 5)	5 (100)	5 (100)	5 (100)	0 (0.0)	0 (0.0)	0 (0.0)	0 (0.0)	0 (0.0)	0 (0.0)	0 (0.0)
Total (n= 243)	231 (95.1)	222 (91.4)	221 (91.0)	134 (55.1)	128 (52.7)	125 (51.4)	16 (6.6)	15 (6.2)	4 (1.6)	0 (0.0)

*AMP* ampicillin, *CTX* cefotaxime, *CFX* cefoxitin, *CP* chloramphenicol, *SM* streptomycin, *SUL* sulfamethoxazole, *OTC* oxytetracycline, *KM* kanamycin, *OFLX* ofloxacin, *CTF*, ceftiofur

Table 4 shows the prevalence and distribution of different multidrug resistance on each serovar: On overall, 231 *Salmonella* strains investigated were resistant or intermediately resistant to three or more of the 10 antimicrobial agents tested. Resistance to three antimicrobials was detected in 91 (37.4%) of the isolates. Resistance to four, five and six antimicrobials were detected 18 (7.4%), 9 (3.7%) and 104 (42.8%), respectively.

**Table 4:** Prevalence and distribution of different multidrug resistance phenotypes among three serovars isolated

Serovars	Resistance pattern									Total
	0	1	2	3	4	5	6	7	8	
*S.* Infantis	5	3	4	78	17	8	19	5	1	140
*S.* Manhattan	–	–	–	8	1	1	85	3	–	98
*S.* Schwarzengrund	–	–	–	5	–	–	–	–	–	5
Total	5	3	4	91	18	9	104	8	1	243

Resistance pattern of 3: SSuT

Resistance pattern of 4: ASSuT

Resistance pattern of 6: ASSuT-CT, CF and ASSuT-CT, CX

*S* Streptomycin, *Su* Sulfamethoxazole, *T* Oxytetracycline, *A* Ampicillin, *CT* cefotaxime, *CF* Ceftiofur, *CX* Cefoxitin

*S.* Infantis has a variety of resistance to antimicrobials agent examined from sensitive to resistance to eight antimicrobials: 5 strains were sensitive, resistance to three antimicrobials was highest 78 (55.7%), followed by resistance to six, four and five antimicrobials agent at 19 (13.6%); 17 (12.1%) and 8 (5.7%) strains, respectively. Multidrug resistance to more than six antimicrobials was detected in 113 (46.5%) of the isolates, and most of them were *S.* Manhattan (88/113), all 5 strains of *S.* Schwarzengrund have three resistance pattern (SSuT).

In Table 5, we have described the resistance proportion of *S.* Infantis and *S.* Manhattan to ampicillin, cefotaxime, ceftiofur and cefoxitin for each year during the period 2009–2012. In the course of the four years of study, resistance proportion of *S.* Manhattan to ampicillin, cefotaxime and ceftiofur (from 76.0% for ceftiofur in 2009 to 100% for three different antibiotics in 2012) was much higher than in *S.* Infantis (7.9% for ceftiofur in 2012 to 52.3% for ampicillin in 2010). On the other hand, all the strains of *S.* Manhattan (98) were sensitive to cefoxitin, while 10.7% (15/140) of *S.* Infantis were resistant to cefoxitin in the period of study.

**Table 5:** The proportion of *S.* Infantis and *S.* Manhattan resistance to ampicillin, cefotaxime and ceftifuor in each year from 2009 to 2012

Year (No. isolated)	S. Infantis				Year (No. isolated)		S. Manhattan		
	AMP (%)	CTX (%)	CTF (%)	CFX (%)		AMP (%)	CTX (%)	CTF (%)	CFX (%)
2009 (35)	13 (37.1)	12 (34.3)	12 (34.3)	4 (11.4)	2009 (14)	14 (100)	13 (92.9)	13 (92.9)	0 (0.0)
2010 (35)	19 (52.3)	16 (45.7)	15 (42.9)	6 (17.1)	2010 (25)	20 (80.0)	20 (80.0)	19 (76.0)	0 (0.0)
2011 (32)	5 (15.6)	5 (15.6)	5 (15.6)	3 (9.4)	2011 (32)	32 (100)	32 (100)	32 (100)	0 (0.0)
2012 (38)	4 (10.5)	3 (7.9)	3 (7.9)	2 (5.3)	2012 (27)	27 (100)	27 (100)	27 (100)	0 (0.0)
Total (140)	41 (29.3)	36 (25.7)	35 (25.0)	15 (10.7)	Total (98)	93 (94.9)	92 (93.9)	91 (92.9)	0 (0.0)

*AMP* ampicillin, *CTX* cefotaxime, *CTF* ceftiofur, *CFX* Cefoxitin

To understand the changes in the antimicrobial resistance of the *Salmonella* isolates over time, we compared our results to data obtained in two previous studies [14, 15]. The comparison is shown in Table 6. In all three studies, all *Salmonella* strains were susceptible to chloramphenicol, with a MIC ≥ 32 μg/mL. The percentage of antimicrobial-resistant strains was also high for three other antibiotics, streptomycin, sulfamethoxazole, and oxytetracycline, although rates of resistance decreased significantly over time (*p* < 0.05).

**Table 6:** Antimicrobial susceptibility profiles in this study and previous studies of *Salmonella* isolates from broiler chickens in Japan

Antimicrobial agent	MIC break-point (μg/mL)	No. of resistant isolates (%)		
		Previous studies		This study
		2004–2006 (*n* = 120)^a^	2007–2008 (*n* = 93)^b^	2009–2012 (*n* = 243)^c^
SM	≥16	120 (100)	86 (92.5)	231 (95.1)
OTC	≥16	120 (100)	86 (92.5)	222 (91.4)
SUL	≥512	120 (100)	86 (92.5)	221 (91.0)
AMP	≥32	29 (22.4)	34 (36.5)	134 (55.1)
CTX	≥4	11 (9.1)	33 (35.5)	128 (52.7)
KM	≥64	9 (7.5)	12 (12.9)	16 (6.6)
CFX	≥32	0 (0.0)	8 (8.6)	15 (6.2)
OFLX	≥2	25 (20.8)	11 (11.8)	4 (1.6)
CP	≥32	0 (0.0)	0 (0.0)	0 (0.0)

*AMP* ampicillin, *CTX* cefotaxime, *CFX* cefoxitin, *CP* chloramphenicol, *SM* streptomycin, *SUL* sulfamethoxazole, *OTC* oxytetracycline, *KM* kanamycin, *OFLX* ofloxacin

^a^Cited from [15]

^b^Cited from [14]

^c^This study

*Significantly increased from the period of 2004–2006 (*p* < 0.05)

# Significantly increased from the period of 2007–2008 (*p* < 0.05)

^$^Significantly decreased from the period of 2004–2006 (*p* < 0.05)

In all three studies, we observed a significant increase in the rates of antibiotic resistance for two antibiotics, ampicillin and cefotaxime (*p* < 0.05). For ampicillin, the resistance rate was 22.4% in 2004–2006, 36.5% in 2007, and 55.1% in the present study period (2009–2012). A similar trend was observed for cefotaxime resistance, with 9.1% in the first study period, 35.5% in the second study period, and 52.7% in 2009–2012.

In contrast, the percentage of ofloxacin-resistant strains decreased dramatically (and significantly) with time across the three study periods, from 20.8% in 2004–2006 to 11.8% in 2007 and 2008, and 1.6% in the present study (*p* < 0.05).

## Discussion

The overall percentages of *Salmonella-*positive flocks (70.6%) and samples (14%) detected in 1998–2003 [16] were higher than the corresponding percentages in 2004–2012, which is clearly demonstrated in the studies by Shahada et al. [15] and Chuma et al. [14] as well as the present study (Table 1). In 2004–2006, the percentage of positive flocks was 39.2% and the percentage of positive samples was 5.2% [15]. In 2007 and 2008, the respective positive rates were 58.7 and 6.3% [14]. This decline in the percentages of positive flocks and samples described above may be due to the use of certain antibiotics in chicken production in Kagoshima, Japan in 2004–2012.

The prevalence of *Salmonella* in Kagoshima, Japan observed in the present study (shown in Table 1) was similar to that reported in the Kyushu region of Japan, where 88.0% of cecal samples from broiler flocks were positive for *Salmonella* [17]. This same rate of positivity for *Salmonella* infection in the flock in the two studies may be because the studies were conducted in the regions with the same climatic conditions.

The serovar changes among *Salmonella* isolates were very clear when the present serovars were compared to those in previous studies. In 1998–2003, 93.4% (526/563) of isolates originating from 135 flocks were *S.* Infantis [16]. In 2004–2006, 100% (193/193) of *Salmonella* isolates were *S.* Infantis, whereas in 2007 and 2008, 97.4% (113/116) of *Salmonella* isolates were *S.* Infantis, and 2.6% (3/116) were *S.* Manhattan [2].

Our result demonstrated the same predominant *Salmonella* serovars (as shown in Fig. 1) as in the other previous studies conducted in Kyushu, Japan. However, there were some differences; for instance, of the 184 *Salmonella* strains isolated from broiler chickens, 123 were *S.* Schwarzengrund (O4:d:1,7), 41 were *S.* Infantis (O7:r:1,5), 9 were *S.* Manhattan (O6,8:d:1,5), 3 were *S.* Yovokome (O8:d:1,5), 5 were OUT:d:1,7, and 4 were OUT:r:1,5 [14].

To our knowledge, this is the first study examining serovar changes in *Salmonella* isolates from broiler chickens over time in Japan. In this study, only three serovars, *S.* Manhattan, *S.* Infantis, and *S.* Schwarzengrund (Fig. 1), were detected. This result is similar to those reported in other countries in Asia (Hyun-Jung [18, 19]), with *S.* Infantis as the main serovar. Although, the main serovar in some European countries was *S.* Typhimurium (Terentjeva et al. [20], [21], Wierup et al. [22]). The different *Salmonella* serovars present in broilers may depend on the region or country due to variations in climate, geographical regions and chicken husbandry practices among countries.

This is also first study to report the relationship between serovar and antimicrobial resistance in *Salmonella* isolates from broiler chickens in Kagoshima, Japan. In the previous studies, these results were not clearly presented. In China, 457 *Salmonella* isolates from chickens, pigs, and dairy cows were most commonly resistant to nalidixic acid (39.17%), sulfamethoxazole-trimethoprim (39.61%), doxycycline (28.22%), and tetracycline (27.58%) [23]. A study in Serbia showed that 100% of *S.* Infantis isolates were resistant to ciprofloxacin and anlidixic acid [24]. In Kagoshima (2007–2008) most *Salmonella* isolates were *S.* Infantis; however, the rate of resistance to ofloxacin was only 11.8% [14]. In addition, a study in Iran reported high fluoroquinolone resistance in both *S.* Infantis and *S.* Enteritidis isolates [19]. The low fluoroquinolone resistance rates in our study may be explained by the differences in the serovars of *Salmonella* isolates. There appeared to be a relationship between the serovars of the *Salmonella* isolates and antimicrobial resistance, especially for ampicillin, cefotaxime, and ofloxacin.

Increased multidrug resistant (MDR) has been reported in *Salmonella* isolates in many countries. In the study the high level of MDR observed among *Salmonella* serovars (Table 4) were in agreement with several studies from different countries [19, 23, 25]. Special *S.* Infantis has wide resistance pattern from zero to eight in total 10 antimicrobials agent were tested.

From Tables 3, Table 5 and Fig. 1. It can be inferred that the resistance rate of *Salmonella* to certain antibiotics (streptomycin, sulfamethozaxole, oxytetracycline, chloramphenicol and ofloxacin) remained unchanged each year during the period 2009–2012. However, the proportion of serovars isolated each year varied. All three isolated serovars were sensitive to chloramphenicol and ofloxacin but resistance to streptomycin, sulfamethozaxole, and oxytetracycline. Since, the number isolated strains of *S.* Schwarzengrund was very small (5 strains isolated during 2009–2012), it might not be expected to show changes in antimicrobial resistance rate with each year.

In the present study, the percentage of resistance to ampicillin, cefotaxime and ceftiofur showed a huge difference between *S.* Manhattan and *S.* Infantis. The resistance of *S.* Manhattan to the three antibiotics mentioned above was more than 3 times higher than in *S.* Infantis in the hold period. In particular, during 2010–2012, the resistance rate of *S.* Infantis decreased while an opposite trend was observed for *S.* Manhattan; the resistance rate of *S.* Manhattan increased eventually to 100%. This indicates a great influence of serovar change on the resistance to the three antibiotics (ampicillin, cefotaxime and ceftiofur).

In Kagoshima, Japan, certain antibiotics, such as: ampicillin, enrofloxacin, amoxicillin and doxycycline are used in the treatment of broiler ascites caused by *E.coli* infection in broiler chicken. That might have affected to the rate of antimicrobial resistance to similar antibiotics, such as β-lactam, tetracycline, in case of *Salmonella*.

In the previous reports from 2007 to 2008, the prevalence rates of *S.* Infantis and *S.* Manhattan were 97.4 and 2.6%, respectively, and the rates of penicillin, cefotaxime, and ofloxacin resistance were 36.5, 35.5, and 11.8%, respectively. In 2004–2006, when all isolates were *S.* Infantis (100%), the rates of penicillin, cefotaxime, and ofloxacin resistance were 22.4, 9.1, and 20.8%, respectively [14]. In our survey, the percentage of *S.* Manhattan isolates resistant to ampicillin and cefotaxime was higher than in *S.* Infantis. From these results, it seems that the increase in the proportion of the *S*. Manhattan serovar leads to the increase in resistance to ampicillin, cefotaxime and ceftiofur with each passing year; this observation is supported by data from the present study and from previous studies.

## Conclusions

Taken together, our data show that there was no significant change in the prevalence of *Salmonella* in broiler chickens in Kagoshima, Japan in 2009–2012 when compared to the prevalence in previously surveyed time periods. We have not yet found a definitive pattern in the prevalence of *Salmonella* and the rates of resistance to some antibiotics. However, when the proportion of *S.* Manhattan isolates increased, the percentage of penicillin-, cefotaxime- and ceftifour-resistant isolates showed a similar increasing trend. Furthermore, the percentage of ofloxacin-resistant strains decreased when the percentage of *S.* Infantis isolated decreased.

Continuous research on the relationship between *Salmonella* isolates serovars and the antimicrobial resistance profiles of each serovar will help reduce the risk of antimicrobial resistant organisms.

## Methods

### Sampling

We analyzed a total of 3071 cecal specimens derived from 192 broiler flocks (ca. 10.000 birds per flock) collected by prefectural officials at an accredited poultry processing plant during the period 2009–2012. The poultry processing plant released these samples (which would otherwise have been disposed of as waste material) with the approval of prefectural officials and sent them to our laboratory. Typically, 16 randomly selected samples per flocks were collected fortnightly [14].

### Salmonella isolation and identification

Samples were collected using sterile techniques, placed in sterile plastic sampling bags, and chilled with ice blocks during transport. Samples were delivered to the Laboratory of Veterinary Public Health, Kagoshima University, and cultured on the day of arrival. Approximately 1 g of cecal contents was aseptically mixed with 5 mL of sterilized distilled water and homogenized by vortexing. Then, 1 mL of the suspension was pre-enriched in 5 mL of Hajna tetrathionate broth (Eiken Chemical Co., Ltd., Tokyo, Japan) and incubated in a water-bath at 42 °C. After 24 h incubation, a loopful of the culture was streaked onto a selective Rambach agar plate, which was incubated at 37 °C for 24 h [16].

Suspected pink colonies were selected from each plate and streaked on nutrient agar slants. Salmonella identification was confirmed by biochemical tests, including fermentation of glucose, lactose and sucrose, hydrogen sulfide production, citrate utilization, lysine decarboxylation, methyl red and indole tests. Serotyping of isolated *Salmonella* strains was performed with reliable commercial antisera (Difco, Detroit, MI, USA), and the results were interpreted according to the Kaufmann-White scheme [26].

### Determination of minimum inhibitory concentrations (MICs)

The antimicrobial susceptibility of the *Salmonella* isolates was assessed by the agar dilution method on Mueller-Hinton agar (Oxoid Ltd., Basingstoke, UK) plates according to the guidelines of the Clinical and Laboratory Standards Institute (formerly the National Committee for Clinical Laboratory Standards [NCCLS]) [27]. Strains were tested for sensitivity to ampicillin, chloramphenicol, streptomycin, sulfamethoxazole, oxytetracycline, kanamycin, ofloxacin, cefotaxime, cefoxitin, ceftiofur. The MIC range was set at 0.25–512 μg/mL for all tested antimicrobial agents. MIC breakpoints were interpreted according to the new criteria established by the Clinical and Laboratory Standards Institute [28, 29]. *Escherichia coli (E. coli)* ATCC 25922 and *Staphylococcus aureus* ATCC 29213 were used as quality control strains.

### Statistical analysis

The prevalence of antimicrobial-resistant isolates across three study periods was compared by using multiple comparisons. A chi-square test was first performed to detect significant differences for each antimicrobial agent. When the result was significant, a test for multiple comparisons of proportions [30] was then performed.

## Abbreviations

AMP: Ampicillin
CFX: Cefoxitin
CP: Chloramphenicol
CTX: Cefotaxime
KM: Kanamycin
MDR: Multidrug resistant
MIC: Minimum inhibitory concentration
No: Number
OFLX: Ofloxacin
OTC: Oxytetracycline
SM: Streptomycin
SUL: Sulfamethoxazole

## References

[1] Kirk MD, Pires SM, Black RE, Caipo M, Crump JA, Devleesschauwer B (2015). World health organization estimates of the global and regional disease burden of 22 foodborne bacterial, protozoal, and viral diseases, 2010: a data synthesis. PLoS Med.

[2] Hedican E, Smith K, Jawahir S, Scheftel J, Kruger K, Birk R (2009). Multistate outbreaks of *Salmonella* infections associated with live poultry – United States, 2007. Morb Mortal Wkly Rep.

[3] Hope BK, Baker AR, Edel ED, Hogue AT, Schlosser WD, Whiting E (2002). An overview of the *Salmonella* Enteritidis risk assessment for shell eggs and egg products. Risk Anal.

[4] Hafez H (2001). *Salmonella* infections in poultry: diagnosis and control. Period Boil.

[5] OMWANDHO Charles O. A., KUBOTA Takayuki (2010). Salmonella enterica serovar Enteritidis: a Mini-review of Contamination Routes and Limitations to Effective Control. Japan Agricultural Research Quarterly: JARQ.

[6] Ministry of Health, Labour and Welfare (MHLW). Statistics of food poisoning in Japan. Available at: http://www.mhlw.go.jp/stf/seisakunitsuite/bunya/kemkou-iryou/shokuhin/syokuchu/04.html Accessed November 4, 2015. Japanese.

[7] Nógrády N, Király M, Davies R, Nagy B (2012). Multidrug resistant clone of *Salmonella* Infantis of broiler in Europe. Int J Food Microbiol.

[8] Furukawa I, Ishihara T, Teranishi H, Saito S, Yatsuyanagi J, Wada E, Kumagai Y, Takahashi S, Konno T, Kashiro H, Kobayashi A, Kato N, Hayashi K, Fukushima K, Ishikawa K, Horikawa K, Oishi A, Izumiya H, Ohnishi T, Konishi Y, Kuroki T (2017). Prevalence and characteristics of *Salmonella* and *Campylobacter* in retail poultry meat in Japan. Jpn J Infect Dis.

[9] Aarestrup FM, Hendriksen RS, Lockett J, Gay K, Teates K, Mcdermott PF (2007). International spread of multidrug-resistant Salmonella Schwarengrund in food products. Emerg Infect Dis.

[10] Silva KC, Fontes LC, Moreno AM, Astolfi-Ferreira CS, Ferreira AJ, Linconpan N (2013). Emergence of extended-spectrum-beta-lactamase CTX-M-2-producing Salmonella enterica serovars Schwarengrund and Agona in poultry farm. Antimicrob Agents Chemother.

[11] Su L, Chiu C, Chu C, Ou J (2004). Antimicrobial resistance in nontyphoid *Salmonella* serovars: a global challenge. Clin Infect Dis.

[12] Gyles CL (2008). Antimicrobial resistance in selected bacteria from poultry. Anim Health Res Rev.

[13] World Health Organization. *Drug-resistant Salmonella.* http://www.who.int/mediacentre/factsheets/fs139/en/. 2018.

[14] Chuma T, Miyasako D, Dahshan H, Takayama T, Nakamoto Y, Shahada F, Akiba M, Okamoto K (2013). Chronological change of resistance to β-lactams in *Salmonella enterica* serovar Infantis isolated from broilers in Japan. Front Microbiol.

[15] Shahada F, Sugiyama H, Chuma T, Sueyoshi M, Okamoto K (2010). Genetic analysis of multi-drug resistance and the clonal dissemination of β-lactam resistance in *Salmonella* Infantis isolated from broilers. Vet Microbiol.

[16] Shahada F, Chuma T, Tobata T, Okamoto K, Sueyoshi M, Takase K (2006). Molecular epidemiology of antimicrobial resistance among Salmonella enterica serovar Infantis from poultry in Kagoshima, Japan. Int J Antimicrob Agents.

[17] Yamazaki W, Uemura R, Sekiguchi S, Dong J-B, Watanabe S, Kirino Y, Mekata H, Nonaka N, Norimone J, Sueyoshi M, Goto Y, Horii Y, Kurogi M, Yoshino S, Misawa N (2016). *Campylobacter* and *Salmonella* are prevalent in broiler farms in Kyushu, Japan: results of a 2-year distribution and circulation dynamics audit. J Appl Microbiol.

[18] Park H-J, Chon J-W, Lim J-S, Seo K-H, Heo E-J, Wee S-H, Sung K, Moon J-S (2015). Prevalence analysis and molecular characterization of *Salmonella* at different processing steps in broiler slaughter plants in South Korea. J Food Sci.

[19] Rahmani M, Peighambari SM, Svendsen CA, Cavaco LM, Agero Y, Hendriksen RS (2013). Molecular clonality and antimicrobial resistance in *salmonella enterica* serovars Enteritidis and Infantis from broilers in three nothern regions of Iran. BMC Vet Res.

[20] Terentjeva M, Avsejenko J, Streikisa M, Utinane A, Kovalenko K, Berzins A (2017). Prevalence and antimicrobial resistance of Salmonella in meat and meat products in Latvia. Ann Agri Environ Med..

[21] El-Sharkawy H, Tahoun A, El-Galiel A, El-Gohary E-AM, El-Khayat M, Gillespie T, Kitade Y, Hafez M, Heinrich N, El-Adawy H (2017). Epidemiological, molecular characterization and antibiotic resistance of *Salmonella enterica* serovars isolated from chicken farms in Egypt. Gut Pathogens.

[22] Wierup M, Wahlstrom H, Lahti E, Eriksson H, Jansson DS, Odelros A, Ernholm L. Occurrence of Salmonella spp.: a comparison between indoor and outdoor housing of broilers and laying hens. Acta Vet Scand. 2017;59:13. DOI 10.1186/s13028-017-0281-4. 10.1186/s13028-017-0281-4 PMC5320760 28222764

[23] Kuang X, Hao H, Dai M, Wang Y, Ljaz Ahmad L, Liu Z, Yuan Z (2015). Serovars and antimicrobial susceptibility of Salmonella spp isolated from farm animals in China. Front Microbiol.

[24] Velhner M, Kozoderovi G, Grego E, Gali N (2014). Clonal spread of *Salmonella enterica* serovar Infantis in Serbia: acquisition of mutations in the topoisomerase genes *gyrA* and *parC* leads to increased resistance to fluoroquinolones. Zoonoses Public Health.

[25] Nógrády N, Kados G, Bistyak A, Turcsányi I, Mészaros J, Galántai Z, Juhász A, Samu P, Kaszanyitky JE, Pászti J, Kiss I (2008). Prevalence and characterization of *Salmonella* Infantis isolates from different points of the broiler chicken-human food chain in Hungary. Int J Food Microbiol.

[26] Popoff MY (1992). Antigenic formulas of the *Salmonella* serovars. WHO Collaborating Centre for Reference and Research on *Salmonella*.

[27] Shahada F, Amamoto A, Chuma T, Shirai A, Okamoto K (2007). Antimicrobial susceptibility phenotypes, resistance determinant and DNA fingerprints of *Salmonella enterica* serovar typhimurium isolated from bovine in southern Japan. Int J Antimicrob Agents.

[28] Clinical and Laboratory Standards Institute. Performance Standards for Antimicrobial Susceptibility Testing: 22th Information Supplement M100-S22, Wayne, PA. 2012.

[29] Clinical and Laboratory Standards Institute. Performance Standards for Antimicrobial Disk and Dilution Susceptibility Test for Bacteria Isolated From Animals. Approved Standard-Fourth Edition M31-A4. 2013. Vol 33. No 7.

[30] Ryan TA (1960). Significance tests for multiple comparison of proportions, variances, and other statistics. Psychol Bull.

